# A Comparison of Near-Infrared Autofluorescence Findings in Benign Versus Malignant Adrenal Tumors

**DOI:** 10.1245/s10434-024-15430-6

**Published:** 2024-05-27

**Authors:** Panagiotis Bletsis, Ege Akgun, Gustavo Romero-Velez, Anibal La Riva, Eren Berber

**Affiliations:** Department of Endocrine Surgery, Metabolism and Endocrinology Institute, Cleveland Clinic Foundation, Cleveland, OH USA

## Abstract

**Background:**

Many adrenal tumors are deemed radiologically indeterminate and surgically removed. Adrenal tissue, like parathyroid glands, exhibits near-infrared autofluorescence (NIRAF) properties. This study was designed to investigate the potential of NIRAF to differentiate benign versus malignant adrenal tumors.

**Methods:**

Patients undergoing adrenalectomy between October 2021 and May 2023 were prospectively studied. Adrenalectomy specimens were inspected with NIRAF imaging. Specimen autofluorescence (AF) characteristics were recorded. Comparisons were made between different tumor types and a logistic regression model was constructed to differentiate benign versus malignant tumors. A receiver operating characteristic curve was used to identify an optimal AF threshold differentiating benign versus malignant tumors.

**Results:**

A total of 108 adrenal specimens were examined: adrenocortical adenomas/other benign lesions (*n* = 72), pheochromocytomas (*n* = 18), adrenocortical neoplasms of uncertain behavior (*n* = 4), and malignant tumors (*n* = 14). A significant difference in normalized AF intensity was identified when comparing adrenocortical adenomas (3.08 times background) with pheochromocytomas (1.95, *p* = 0.001) and malignant tumors (1.11, *p* < 0.0001). The Area Under the Curve differentiating benign vs malignant tumors was 0.87, with an optimal normalized AF threshold at 1.93.

**Conclusions:**

Different adrenal pathologies exhibit diverse AF properties. These findings suggest a potential intraoperative utility of NIRAF in predicting benign versus malignant nature for radiologically indeterminate adrenal tumors.

The frequency of incidental adrenal masses detected on unrelated imaging has risen with increasing number and quality of cross-sectional abdominal imaging.^1^ The clinical approach to adrenal masses aims to characterize the type of adrenal tumor by comprehensive assessment of patient and family history, biochemical profile to determine functionality, and radiographic features such as size, growth, architecture, borders, Hounsfield unit (HU) density, and relationship to surrounding structures.^1–3^ The treatment algorithm for functional tumors is relatively straightforward, with surgical resection recommended in most cases. However, for nonfunctional tumors, the decision-making process can become more complex, particularly when masses are lipid poor (> 10 HU) and have absolute and relative washouts of < 60 and < 40% respectively.^2–5^ These radiographically indeterminate masses most often undergo adrenalectomy, especially when there are high risk clinical features on imaging, such as heterogeneity, growth over time, or patient history of extra-adrenal cancer.^2^

Minimally invasive adrenalectomy (laparoscopic/robotic transabdominal or retroperitoneal approaches) is the most common approach for all benign and select malignant adrenal tumors.^6,7^ Intraoperative surgical technique is modified for benign versus malignant tumors—the latter requires a wider resection to ensure negative margins and decrease the risk of recurrence. Currently, ultrasound and indocyanine green (ICG) fluorescent imaging are the only available intraoperative modalities to provide surgeons with information regarding adrenal identification, delineation of gland/tumor borders, and location of critical vascular structures during minimally invasive adrenalectomies.^8–10^ This is particularly helpful in challenging cases where there is an overabundance of retroperitoneal fat present obscuring the anatomy and when it is crucial to ensure removal of all adrenal tissue based on the pathology of disease being treated.^11^ However, ultrasound and ICG each have their own inherent limitations.^11–13^

Recent studies have shown that adrenal tissue, particularly the adrenal cortex, exhibits intense near infrared autofluorescence (NIRAF) properties similar to parathyroid tissue, introducing the prospect of a new adjunct to assist dissection in adrenalectomies.^11–15^ The purpose of this study was to determine whether NIRAF could be used to differentiate benign versus malignant adrenal tumors during adrenalectomy.

## Methods

This was an institutional review board-approved prospective study of patients at a single tertiary center undergoing minimally invasive adrenalectomy by the senior author between October 2021 and May 2023. Decision for adrenalectomy was based on the American Association of Endocrine Surgeons and the American Association of Clinical Endocrinologist guidelines for the management of adrenocortical tumors.^2,16^ The surgical indications for adrenalectomy included hormone secreting tumors (primary hyperaldosteronism, pheochromocytoma, mild autonomous cortisol secretion, and Cushing’s syndrome), radiologically indeterminate nonsecreting tumors, and radiologically suspicious tumors of primary or metastatic malignant potential taken in the clinical context of the patient’s medical history and risk factors.

In all procedures, resected surgical specimens were inspected with near-infrared fluorescence imaging using 750-nm camera (Fluobeam LX) 15-20 cm above the specimen on the back table. The fluorescence measurements were only taken from the anterior surface of the tumor and not from deeper portions. Any patients who received ICG intraoperatively were excluded from this study, because the injected fluorophore would interfere with the intrinsic NIRAF properties of adrenal tissue. In the cases done open, the camera also was used intraoperatively.

Using baseline data from our initial study where approximately 80% of the tumors were benign adrenocortical adenomas (ACA), in order to differentiate a normalized AF difference of 1 between benign ACA versus pheochromocytomas and malignant tumors, a sample size of at least 50 was required.^11^ Autofluorescence (AF) characteristics of the adrenal tumor, normal adrenocortical parenchyma, and surrounding retroperitoneal fat were analyzed from recorded video and still images using ImageJ software (National Institutes of Health, Bethesda, MD).

Tumor AF was reported as a normalized intensity ratio of the tumor AF intensity compared with the background blue surgical drape AF intensity. Using the ImageJ software (National Institutes of Health, Bethesda, MD), the edges of the intact anterior portion of the tumor, where little if any periadrenal tissue is present were drawn, the AF intensity was recorded and divided by the AF intensity of a 1-cm × 1-cm circle of the background drape. All statistical analyses were performed with SAS version 9.4 (SAS, Cary, NC). The probability distribution of the normalized AF was assessed by using the Kolmogorov-Smirnov test. Because these were noted to have a non-normal distribution, the comparison between groups was done with non-parametric tests. The Wilcoxon rank-sum and Kruskal-Wallis test were used for two and multiple-group comparisons, respectively. After excluding cases of pheochromocytoma, in which the diagnosis is made biochemically, a binary logistic regression model using the normalized AF was done to differentiate malignant vs benign. The performance of the model was assessed with the area under the receiver operating curve (ROC). The ideal normalized AF threshold to differentiate malignant versus benign was then calculated based on the Youden Index from the ROC. Statistical significance was considered at *p* < 0.05. Unless otherwise indicated, results are reported as counts/percentages for categorical variables and median/interquartile ranges (IQR) for continuous variables.

## Results

A total of 108 adrenal specimens from 107 patients were examined during the course of the study. The final pathology of the specimens included 69 ACAs, 3 other benign lesions, 18 pheochromocytomas, 4 adrenocortical neoplasms of uncertain malignant potential, and 14 malignant tumors (including adrenocortical carcinomas [*n* = 4], and metastatic tumors [*n* = 10]). Table 1 summarizes the demographic and clinical details of the study patients.

**Table 1:** Demographic and clinical characteristics of study patients

Variable	Value
Age, years	57 (46–66)
Sex	
Female	77
Male	30
Race/ethnicity	
White	94
Black	8
Asian	1
Hispanic	1
Unavailable/declined to answer	3
BMI, kg/m^2^	29 (27–34)
Diagnosis	
Benign	72
Nonsecreting adrenocortical adenoma	16
Cushing’s/mild autonomous cortisol secretion	38
Primary hyperaldosteronism	15
Benign others*	3
Pheochromocytoma	18
Adrenocortical neoplasm of uncertain malignant potential	4
Malignant	14
Metastasis**	10
Adrenocortical carcinoma	4
Radiographically indeterminate nodules	56
Hounsfield units	30 (16–37)
Absolute washout	42 (3–51)
Tumor size, mm	28 (21–42)
Side of tumor	
Left	63
Right	44
Surgical approach	
Robotic	102
Lateral transabdominal	96
Posterior retroperitoneal	7
Open	4
Laparoscopic	1
Operative time, min	137 (114–167)
Length of hospital stay, days	1 (1–1)
90-day complication	
No complication	105
Atelectasis	1
Stroke	1

Continuous data are expressed as median and interquartile range

There are 108 specimens from 107 patients included

*Benign others include cyst (*n* = 1), lymphatic cyst with dystrophic calcification (*n* = 1), ganglioneuroma (*n* = 1)

**Metastatic renal cell carcinoma (*n* = 3), lung adenocarcinoma (*n* = 3), melanoma (*n* = 1), colon adenocarcinoma (*n* = 1), breast adenocarcinoma (*n* = 1), and retroperitoneal sarcoma (*n* = 1)

*BMI* body mass index

**Fig. 1:**
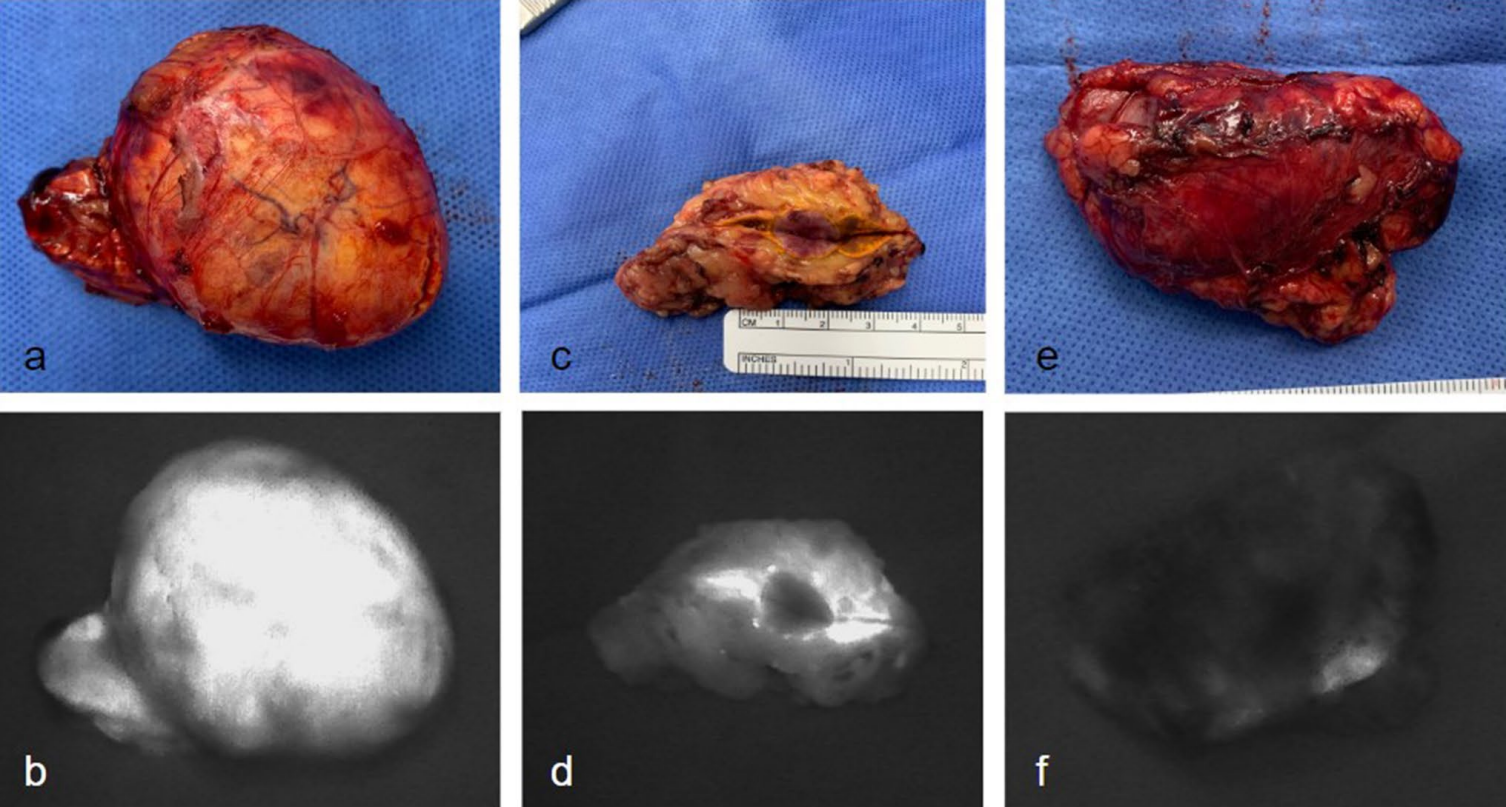
Ex vivo photographs of various specimens and their corresponding AF images. **a** Gross specimen photo of a benign ACA. **b** AF image of a benign ACA. **c** Gross specimen photo of a pheochromocytoma. **d** AF image of a pheochromocytoma. **e** Gross specimen photo of a metastatic renal cell cancer. **f** AF image of a metastatic renal cell cancer

**Fig. 2:**
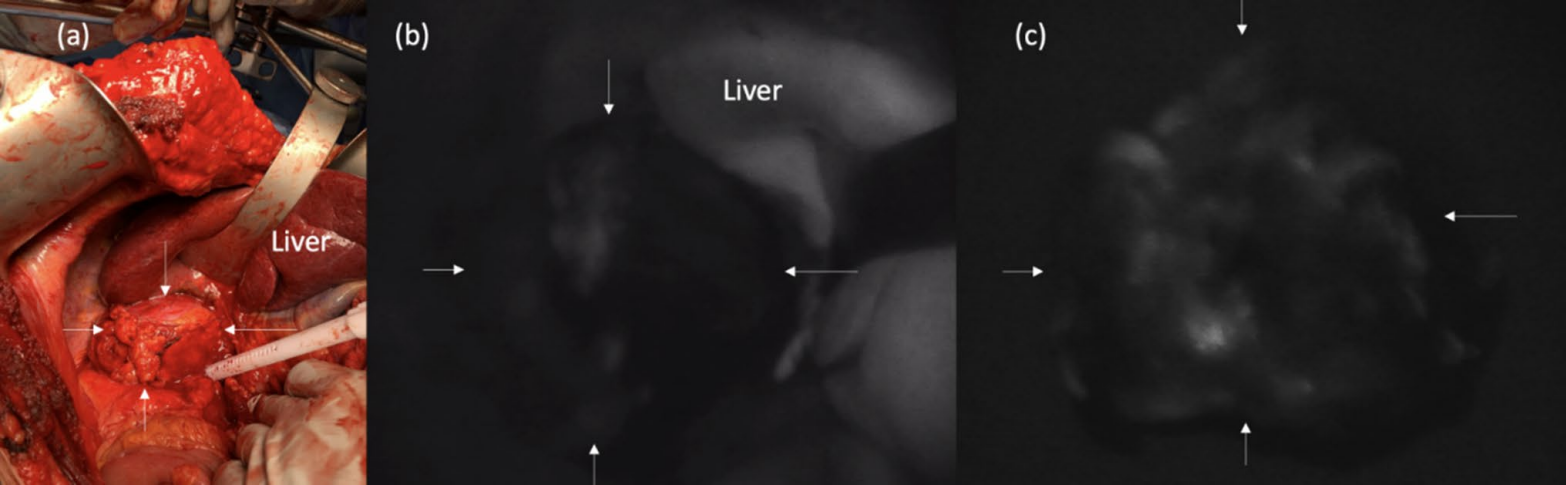
Intraoperative photograph of an adrenal metastasis from renal cell cancer (**a**) with corresponding intraoperative in vivo (**b**) and ex vivo (**c**) AF images. Arrows mark the borders of the tumor

**Fig. 3:**
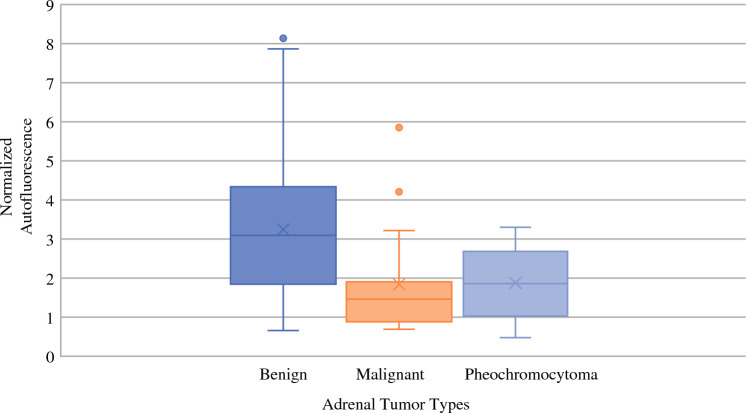
Box plot depicting normalized AF intensities of different adrenal tumor types

Benign adrenocortical tumors demonstrated a brighter AF signal than pheochromocytomas and primary/secondary malignant tumors (Figs. 1, 2). On quantitative analysis, a significant difference in normalized AF intensity was found when comparing benign tumors (3.08 times background [IQR 1.92–4.20]) with pheochromocytomas (1.95 times background [IQR 1.01–2.72], *p* = 0.001) and malignant tumors (1.11 times background [IQR 0.84–1.73], *p* < 0.0001) (Fig. 3). The Area Under the Curve (AUC) to differentiate benign vs malignant tumors was 0.87, with an optimal threshold cutoff normalized AF point at 1.93 for a sensitivity of 93% and specificity of 72%. The positive predictive value (PPV) for predicting a benign tumor was 39%, and negative predictive value (NPV) was 98%. Of all masses, HU density on CT imaging and normalized AF intensity on NIRAF imaging were negatively correlated, with a coefficient of −0.273 (*p* = 0.006). Further analysis was then done on only the radiographically indeterminate adrenal masses. After excluding patients with pheochromocytoma, primary hyperaldosteronism, and all masses with benign characteristics on imaging (HU < 10), there were 56 patients with radiographically indeterminate adrenal masses (38 benign, 14 malignant, 4 adrenocortical neoplasms of uncertain malignant potential). The HU for those were 30 (range 16–37) with a median absolute washout of 42% (3–51%). In this group, benign and malignant tumors had median normalized NIRAF intensities of 2.68 (IQR 1.86–3.87) versus 1.20 (IQR 0.84–1.78) respectively (*p* = 0.0006). When adrenocortical neoplasms of uncertain malignant potential were compared with the malignant tumors, median normalized NIRAF intensities were 4.48 (IQR 2.12–5.58) versus 1.20 (IQR 0.84–1.78), respectively (*p* = 0.0026). Using the cutoff point previously calculated (1.93), the sensitivity was 92%, specificity 74%, PPV 55%, and NPV 97%.

## Discussion

To our knowledge, this is the largest study to date reporting the NIRAF properties of different adrenal tumors. The results of our study strengthen the previously published, yet smaller initial experiences published at three separate academic, high-volume adrenal institutions, including our institution, that benign adrenocortical tumors exhibit significantly more intense AF compared with pheochromocytomas and malignant tumors.^11–13^ In our initial study, the average AF for all benign adrenocortical tumors was 2.23 (*n* = 16), for pheochromocytomas was 0.90 (*n* = 3), and for malignant tumors was 0.90 (*n* = 5).^11^ While the normalized AF values calculated in the current study are slightly higher compared with our initially published experience using the same commercially available NIRAF imaging system, the trend remains the same. The AF ratio of benign adrenocortical adenomas to malignant tumors and pheochromocytomas was 2.48 and 2.48 times more intense in our initial series and 2.77 and 1.58 in our current larger series. We believe the discrepancy is related to the differences in sample sizes. Thomas et al. in their series of 55 patients using another similar commercially available NIRAF imaging system, PDE-Neo II (760-nm camera), found benign adrenocortical tumors to also exhibit more intense AF compared with malignant tumors and pheochromocytomas, with ratios of 1.57 and 1.43 respectively.^12^ Rajan et al., in their series of 22 patients also using the PDE-Neo II camera system, found benign adrenocortical tumors to harbor the most intense AF, with them being 1.50 times more intense than “suspicious/growing” adrenal masses (final pathology not reported to confirm these were in fact malignant tumors) and 1.97 times more intense than pheochromocytomas.^13^ Despite the small series published on adrenal NIRAF, there is concordance seen among three separate groups showing: (1) Adrenal tissue has intrinsic AF properties compared to surrounding tissue both in vivo and ex vivo; (2) Due to these properties, adrenal gland identification and delineation of borders is possible using NIRAF (although this is currently only limited to open cases, because the technology is not compatible with minimally invasive camera systems yet); (3) There are differences in adrenal AF characteristics based on tumor pathology, with benign tumors of adrenocortical origin demonstrating the highest intensity of AF compared with non-cortical tumors (pheochromocytomas and secondary malignant adrenal tumors) and cortical primary malignant adrenal tumors. Because pheochromocytomas can be diagnosed preoperatively with biochemical testing, the utility of AF imaging to help differentiate benign from malignant tumors emerges.

NIRAF technology was initially advocated for parathyroid imaging, with recent studies, including the current one, showing that the adrenal gland exhibits AF in the same spectrum.^11–14^ As with parathyroid tissue, however, the fluorophore responsible for NIRAF in adrenal tissue has not been identified yet, but there may be some commonality between the two, given the increased fluorescence in both of these endocrine organs compared with other tissues studied.^12,15,17^ Due to the AF properties of the adrenal, NIRAF imaging holds promise as an additional intraoperative adjunct for gland identification and delineation of the borders to assist with dissection, similar to ICG, but with the added advantages of not requiring injection/re-injection of dye and lack of allergic complications.^11–13^

Our study’s strength is that we additionally have shown that NIRAF tumor intensity could help to predict which radiographically indeterminate tumors are benign versus malignant intraoperatively, which is a benefit not afforded to the surgeon by either ultrasound or ICG. The limitation of our findings is that the intraoperative NIRAF devices are not compatible with laparoscopic or robotic approaches and can only be used in vivo during open adrenalectomy. Until the technology is developed to integrate with laparoscopic and robotic camera systems, NIRAF detection of adrenal tissues and tumors cannot be truly studied intraoperatively during dissection for minimally invasive adrenalectomies. While in our study the AF of adrenal tumors were normalized against a background drape for consistency across all cases, given the majority of cases (103/107) were performed minimally invasively, the same pattern would be seen in vivo with benign ACA being more autofluorescent than malignant tumors. This is because the retroperitoneum does not have intrinsic AF properties. What would change is the optimal normalized AF value of 1.93, which we calculated as the cutoff for best predicting benign versus malignant tumors. That value can only meaningfully be applied when using the same Fluobeam LX imaging system and technique measuring AF values 15–20 cm above the specimen ex vivo on the back table. Hopefully, our promising findings will stimulate further interest in bringing NIRAF technology to laparoscopic and robotic camera systems to assist the future adrenal surgeon.

## Conclusions

The adrenal cortex, in addition to parathyroid tissue, also possesses AF properties. By measuring the intensity of signals, cortical adrenal tumors deemed indeterminate preoperatively on imaging may be further differentiated using NIRAF intraoperatively to help predict benign versus malignant pathology based on our model. Intraoperatively, this can currently only be done in open cases. Still, once the technology is integrated into robotic/laparoscopic camera systems, this could be a valuable tool during dissection since the vast majority of adrenalectomies now being performed are minimally invasive. Until then, back table assessment can still be a useful adjunct for preoperatively indeterminate tumors after resection, guiding the surgeon to take additional retroperitoneal margins when the normalized AF intensity of the tumor is low.

## Data Availability

The data that support the findings of this study are not shared due to institutional policy.
